# Proton Therapy Adaptation of Perisinusoidal and Brain Areas in the Cyclotron Centre Bronowice in Krakow: A Dosimetric Analysis

**DOI:** 10.3390/cancers16183128

**Published:** 2024-09-11

**Authors:** Marzena Rydygier, Tomasz Skóra, Kamil Kisielewicz, Anna Spaleniak, Magdalena Garbacz, Monika Lipa, Gabriela Foltyńska, Eleonora Góra, Jan Gajewski, Dawid Krzempek, Renata Kopeć, Antoni Ruciński

**Affiliations:** 1 Cyclotron Centre Bronowice, Institute of Nuclear Physics Polish Academy of Sciences, PL31342 Kraków, Poland; marzena.rydygier@ifj.edu.pl (M.R.);; 2 National Oncology Institute—National Research Institute, Krakow Branch, PL31115 Kraków, Poland

**Keywords:** adaptive treatment planning, computed tomography, intensity modulated proton therapy, pencil beam scanning

## Abstract

**Simple Summary:**

Adaptive proton therapy (APT) is an evolving approach to proton beam scanning treatment planning. We performed dosimetric study on two groups of head and neck (H&N) patients to evaluate the influence of plan adaptation on planning target volume (PTV) and organs at risk (OARs) doses, resulting from the changes in patient anatomy observed in control computed tomography (CT). The adapted treatment plans, which incorporated the changes observed in the control CT images, statistically improved mostly PTV coverage compared to initial plan. Study shows that applying adaptive procedures in clinical workflow may increased efficiency by controlling the proper irradiation of the treated area for H&N cancer patients.

**Abstract:**

Applying a proton beam in radiotherapy enables precise irradiation of the tumor volume, but only for continuous assessment of changes in patient anatomy. Proton beam range uncertainties in the treatment process may originate not only from physical beam properties but also from patient-specific factors such as tumor shrinkage, edema formation and sinus filling, which are not incorporated in tumor volume safety margins. In this paper, we evaluated variations in dose distribution in proton therapy resulting from the differences observed in the control tomographic images and the dosimetric influence of applied adaptive treatment. The data from weekly computed tomography (CT) control scans of 21 patients, which serve as the basis for adaptive radiotherapy, were used for this study. Dosimetric analysis of adaptive proton therapy (APT) was performed on patients with head and neck (H&N) area tumors who were divided into two groups: patients with tumors in the sinus/nasal area and patients with tumors in the brain area. For this analysis, the reference treatment plans were forward-calculated using weekly control CT scans. A comparative evaluation of organ at risk (OAR) dose-volume histogram (DVH) parameters, as well as conformity and homogeneity indices, was conducted between the initial and recalculated dose distributions to assess the necessity of the adaptation process in terms of dosimetric parameters. Changes in PTV volume after replanning were observed in seventeen patient cases, showing a discrepancy of over 1 ${\mathrm{cm}}^{3}$ in ten cases. In these cases, tumor progression occurred in 30% of patients, while regression was observed in 70%. The statistical analysis indicates that the use of the adaptive planning procedure results in a statistically significant improvement in dose distribution, particularly in the PTV area. The findings led to the conclusion that the adaptive procedure provides significant advantages in terms of dose distribution within the treated volume. However, when considering the entire patient group, APT did not result in a statistically significant dose reduction in OARs (α = 0.05).

## 1. Introduction

Radiotherapy is typically planned based on a single computed tomography (CT) scan, assuming that the patient’s anatomy and its position relative to the radiation delivery system remain unchanged within predefined limits during the treatment course. Most random uncertainties are addressed by applying safety margins around the target volume. However, certain factors are not entirely considered in the planning target volume (PTV), such as substantial changes in patient anatomy due to variations in tumor volume or fluctuations in organ filling [1,2]. Some of these anatomical changes result from the treatment itself, such as tumor shrinkage, while others may be unrelated to radiation therapy. In general, changes in patient anatomy during proton therapy are likely the most significant source of range uncertainty, as the dose distribution from the proton beam is sensitive to variations in range caused by density changes along the beam path [3].

The goal of adaptive proton therapy (APT) is to ensure the precise delivery of the prescribed dose while adhering to constraints on healthy tissues, particularly if patient geometry undergoes changes throughout a fractionated treatment. There are generally two strategies in adaptive treatment: online and offline adaptation [4]. Online APT can reduce the safety margins by conducting a daily observation of patient anatomy and organ localization. This requires well-verified implementation of online plan reoptimization and validation protocol, which is a clinically difficult approach. The offline adaptation can be implemented using patient control imaging, but the new plan preparation needs additional workload and does not account for the daily patient variations. In general, the APT aims to improve the precision of radiation therapy by systematically monitoring variations in patient anatomy and integrating them into the treatment plan through reoptimization during the course of treatment. A significant challenge in offline plan adaptation is the demanding clinical workflow and the need of additional clinical resources. This process involves imaging, contour definition, plan evaluation, plan adaptation and, ultimately, plan verification [2]. Therefore, the workflow of APT must be optimized to balance potential benefits for the patient against the required resources engaged in this process. Recently, simulation studies were also presented for partial APT, which is an example of the time-optimized solution, but still with the need of clinically approved software for plan preparation and verification, which is not yet available [5].

Different solutions are proposed for APT by research groups and consortiums around the world, such as ProtOnART [6] and RAPTOR [7]. They work on solutions to bring near-real-time APT utilizing Monte Carlo methods, deep learning- or model-based solutions, such as normal tissue complication probability [8,9,10].

In this paper, we present the clinical APT workflow for sinus and brain patients treated at the Cyclotron Centre Bronowice (CCB) Krakow proton therapy center. We hypothesize that APT improves dose distributions delivered to the patient throughout the treatment course. To verify this, we conduct a retrospective dosimetric analysis comparing the dose distributions in the target and organs at risk (OARs), using initial treatment plans recalculated on control CT scans and reoptimized plans after APT.

## 2. Materials and Methods

At the CCB, protons are generated and accelerated to therapeutic energies up to 230 MeV in the isochronous cyclotron C 230 (IBA, Louvain-la-Neuve, Belgium). Additionally, the CCB is equipped with two rotating gantries featuring modern proton pencil beam scanning (PBS) nozzles. Proton radiotherapy procedures performed at the CCB are conducted in collaboration with the National Oncology Institute (Kraków, Poland).

Approximately 12% of patients treated with protons at the CCB between 2020 and mid-2024 underwent APT. The overall statistics of patients treated at CCB with proton beam scanning by year are included in Table 1. The table also provides information on how many patients underwent the APT procedure each year. The majority of APT was performed for tumors located in head and neck (H&N) area (>90%). Usually, control CT scans are obtained weekly or in response to specific changes in anatomy due to drug administration or weight loss. Follow-up CT scans are further used for APT if necessary. Typically, it takes a few days from acquiring a control CT image to delivering the clinically approved adapted plan. This adaptive approach enhances dose conformity in the case of interfractional anatomical variation. However, this method proves insufficient for non-anatomical patient variations, such as organ cavity fillings appearing after the scan and not being detected in the control CT image.

### 2.1. Data

In the CCB Krakow proton center, two types of imaging are conducted. Two-dimensional X-ray imaging is performed daily for the purpose of patient positioning based on bony anatomy. However, these 2D images do not provide information on soft tissue and possible anatomy changes that may be an indication for APT. Therefore, the clinical imaging protocol includes weekly 3D kV CT imaging that provides comprehensive information about both bony structures and soft tissues.

Examination of a patient’s anatomical conditions using the CT scanner is conducted by radiation therapists (RTTs) under the supervision of the physician. The CCB employs a 64-slice Siemens Somatom Definition AS Open (Siemens Healthcare, Erlangen, Germany) for performing CT scans. For H&N localization, the CT scan parameters include a 120 kV X-ray tube voltage, a slice thickness of 1.2 mm and a reconstruction diameter, i.e., the field of view (FoV), of 350 mm.

The subsequent step in preparing the treatment plan involves defining the contours of the PTV with appropriate margins, as well as delineating the OARs. The PTV is created by extending the clinical treatment volume (CTV) by 3 mm to accommodate variations in patient anatomy and positioning. It also considers the uncertainty of the CT calibration curve, equivalent to a 3.5% safety margin of the maximal beam range plus 1 mm.

For each patient treated at the CCB, a planning CT is available with corresponding contours outlined by a radiation oncologist. An intensity modulated proton therapy (IMPT) plan is then optimized for each patient using the Eclipse (v16.1, Varian, Palo Alto, CA, USA) treatment planning system. The proton beam specifications are based on the IBA dedicated scanning nozzle beam model, with the spot sigma (σ) in air ranging from 2.5 mm to 6.4 mm for the applicable nominal beam energies (between 226.1 MeV and 70 MeV, respectively). Dose calculations and optimization during treatment planning are conducted using proton convolution superposition (PCS) or nonlinear universal proton optimization (NUPO), assuming a constant relative biological effectiveness (RBE) equal to 1.1.

A cohort of 21 H&N patients was chosen for analysis (refer to Table A1 and Table A2 from Appendix A). The selection criteria were based on the PTV’s location, requiring replanning due to dose distribution irregularities in the PTV and/or OAR areas. Patients with new immobilizations prepared prior to control CT were not included. Among the 21 patients, 10 had tumors within the sinus or nasal cavity areas, and 11 had brain tumors. The total prescribed dose ranged from 54 to 70 Gy(RBE), depending on the diagnosis. The plans comprised 2–6 fields.

According to the aforementioned tables, the number of days that elapsed from the CT scan to the start of irradiation using the adaptive plan was determined. The average replanning time was 5 (±2) days for patients with tumors in the sinus area and 6 (±4) days for patients with brain tumors. For the entire patient cohort, the average was 5 (±3) days.

### 2.2. Adaptation Protocol

Patients may experience various sources of geometric variation between fractions throughout the course of radiotherapy. Changes in patient anatomy, whether related to treatment or not, may have a dosimetric impact on OARs or lead to dosimetric discrepancies within treatment volume coverage, highlighting the importance of systematic anatomy monitoring [2].

If patient anatomy variations occur, anatomy monitoring may be followed by APT that, however, introduces an additional workload for the staff, mainly because of the necessity of replanning and patient-specific plan quality assurance (QA), and for radiation oncologists who review and approve the plans. Generally, offline adaptation has recently been employed in proton therapy facilities [11].

**Figure 1:**
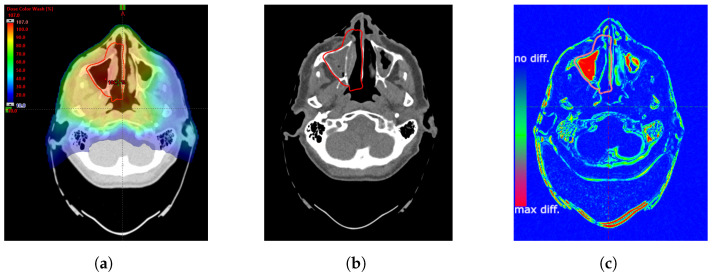
Illustration of changes in sinus filling: (**a**) treatment planning CT overlaid with the initial dose distribution and delineated PTV (red line), (**b**) fusion of the control CT images with the initial PTV outline (red line), (**c**) illustration of a fusion of the initial and control CT images with a color scale highlighting differences: red indicates the greatest difference, while blue signifies no differences.

An offline adaptation is also implemented at the CCB. To preliminarily assess the necessity of APT, a rigid fusion of images from the control scans with the initial scan used for treatment planning is conducted. Changes between the specified scans are analyzed by radiation oncologists and medical physicists. The visual inspection of fused images includes possible variations in the quality of coverage of the treated volume and the exposure of critical organs that may be followed by dose recalculation and evaluation. Exemplary image and contour comparisons are shown in Figure 1.

### 2.3. Plan Recalculation and Data Analysis

**Figure 2:**
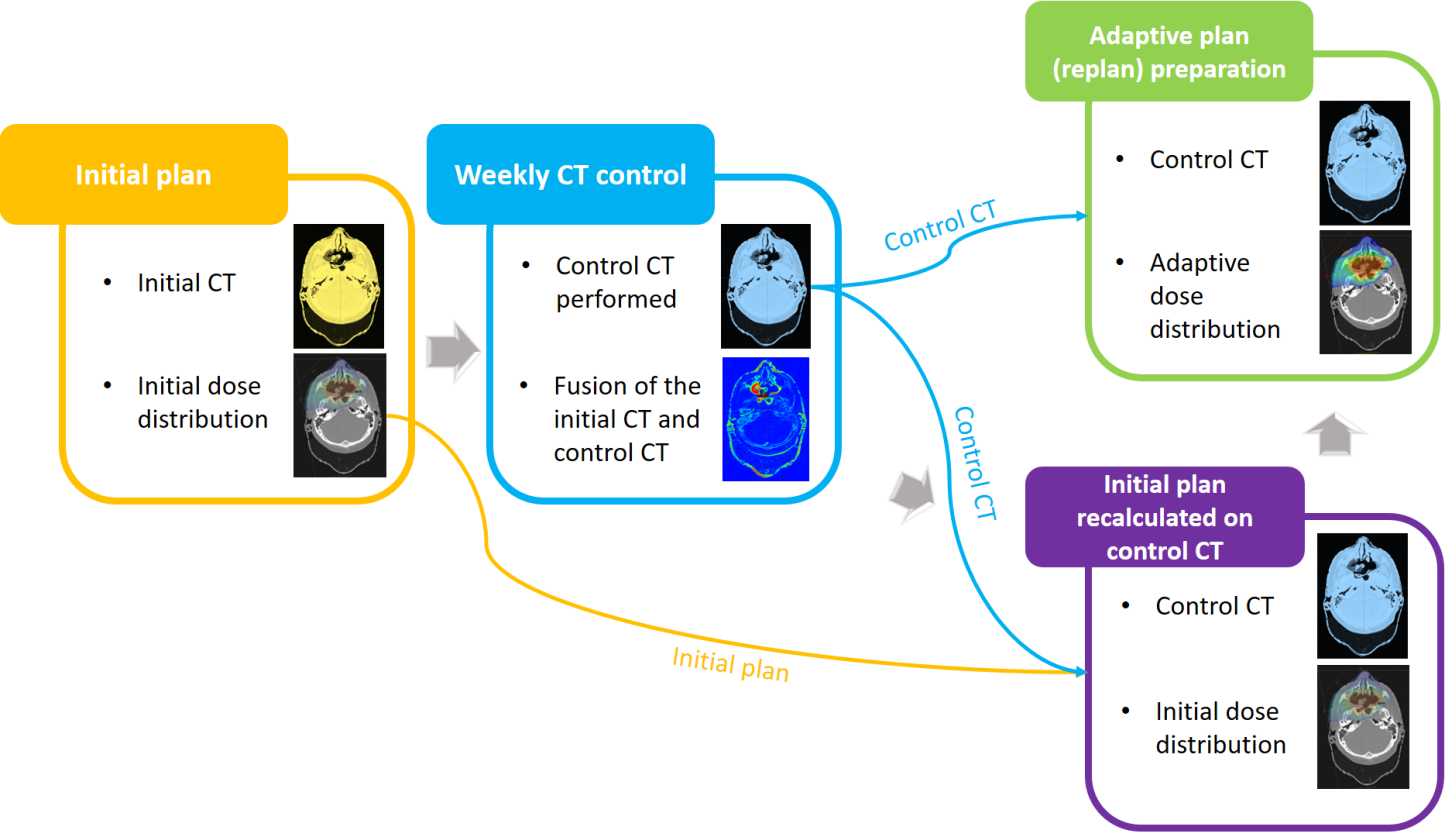
Schematic illustration of the adaptive plan preparation procedure. The yellow box represents the initial plan developed based on the initial CT scans. The blue box illustrates the fusion of the initial and control CT scans. The purple box represents an initial dose distribution recalculated on the control CT scans. The green box represents the newly reoptimized dose calculated on the control CT scans.

To evaluate the dosimetric benefit of APT, the initial treatment plans were forward-calculated on the control CT scans. The analysis involved comparing the originally calculated dose distribution on the control CT scans with the corresponding APT dose distribution. The assessment of all IMPT plans relied on the calculated dose-volume histograms (DVHs) and their associated metrics, i.e., DVH indices. The procedure is illustrated schematically in Figure 2.

The APT aims to achieve the clinical goals established during the development of the initial treatment plan by replanning on the control CT. These goals can be specified for any designated volume, including PTVs and OARs. The comparison of DVH parameters was conducted between adapted plans (control CT, new plan/replan) and non-adapted plans (control CT, initial plan) to illustrate the dosimetric benefits of the adaptations performed in Krakow.

Typically, various constraints for different organs are defined, such as the dose received by percentage of the organ volume (i.e., $D_{98\%}$) or the volume of the organ receiving at least a specific value of the absorbed dose expressed in gray (Gy) or percentage of the prescribed dose (i.e., $V_{95\%}$ or $V_{54\mathrm{Gy}}$). Given the location of the tumors in the selected patient group, structures such as the brain, brainstem, chiasm, optic nerves and spinal cord were chosen for analysis. As these structures correspond to serial organs (organs where disabling any subunit causes the entire organ to fail), parameters such as $D_{\max}$ and $D_{\mathrm{near}-\max}$ (dose delivered to 0.1 ${\mathrm{cm}}^{3}$ of a structure volume) were selected for analysis.

Additionally, parameters of the PTV and metrics describing the quality of coverage of the planned volume were analyzed, including conformity index (CI), homogeneity index (HI) and conformation number (CN):

- The homogeneity index (HI) signifies the slope of the DVH, reflecting the uniformity of the dose distribution within the PTV. The HI is calculated as follows:
  (1) $$\mathrm{HI}=\frac{D_{2\%}-D_{98\%}}{D_{p}}\times 100$$
  where $D_{2\%}$ and $D_{98\%}$ represent the doses received by 2% and 98%, respectively, of the PTV, and $D_{p}$ is the prescribed dose [12]. The ideal value is zero, indicating equality between $D_{2\%}$ and $D_{98\%}$.
- The conformity index (CI) describes the coverage of the PTV with the prescribed dose. The CI is expressed by the following Formula (2):
  (2) $$\mathrm{CI}=\frac{{\mathrm{TV}}_{\mathrm{RI}}}{\mathrm{TV}}$$
  where ${\mathrm{TV}}_{\mathrm{RI}}$ denotes the target volume covered by the reference isodose (e.g., at least 98% of the prescribed dose $D_{p}$), and TV is the target volume. This index varies from 0 (when the entire target volume is located outside 98% $D_{p}$ isodose) to 1 (when the entire target volume receives at least 98% $D_{p}$).
- The conformation number (CN) simultaneously considers the irradiation of the target volume and healthy tissues and is defined as follows:
  (3) $$\mathrm{CN}=\frac{{\mathrm{TV}}_{\mathrm{RI}}}{\mathrm{TV}}\cdot \frac{{\mathrm{TV}}_{\mathrm{RI}}}{V_{\mathrm{RI}}}$$
  where TV is the target volume, $V_{\mathrm{RI}}$ is the volume of the reference isodose (98%). The CN varies from 0 (indicating either a complete lack of conformation or a very large volume of irradiation compared to the target volume) to 1 (in cases when dose coverage is ideal) [13].

In this analysis, eight parameters were assessed [14], with two parameters focusing on OARs in the H&N region ($D_{\mathrm{near}\_\max}$ (0.1 ${\mathrm{cm}}^{3}$), $D_{\max}$). The remaining six parameters characterized the dose distribution and conformity within the PTV area ($D_{\mathrm{mean}}$, $D_{98\%}$, $V_{95\%}$, CI, HI and CN). A summary of the parameter descriptions is provided in Table 2.

A nonparametric statistical hypothesis test, specifically the Wilcoxon signed-rank test, was conducted to compare the parameters of the initial clinical plans before and after the adaptive procedure.

## 3. Results

Table A1 and Table A2 in Appendix A contain a list of patients selected for the study. According to this table, changes in PTV after replanning were observed in seventeen cases, showing a discrepancy of over 1 ${\mathrm{cm}}^{3}$ in ten cases. Each of the investigated patients underwent adaptation only one time. For a change in PTV volume of less than 1 ${\mathrm{cm}}^{3}$, it was assumed that it was a consequence of copying structures onto other CT scans. In cases where the PTV volume exceeded 1 ${\mathrm{cm}}^{3}$, tumor progression occurred in 30% of patients, while regression was observed in 70%. The most significant change in PTV volume was observed in patient #6 of the sinus tumor group (Table A1), reaching 21.4%. In 81% of cases, the motivation behind APT preparation was to enhance the quality of the dose distribution, providing patients with dosimetric benefits such as limiting the dose to critical organs or achieving more optimized PTV dose coverage. Interestingly, in one case, an adaptive plan was devised despite meeting the treatment plan constraints, indicating that APT still improved PTV coverage. The analysis of the results for the patient group reveals that 33.3% of cases necessitated an adaptive plan in the first half of the irradiation cycle, and the remaining 66.7% necessitated an adaptive plan in the second half of the cycle. Table 3 summarizes the timing of the start of irradiation using the adaptive plan during the radiotherapy course for two tumor location groups: sinus/nasal cavity and brain.

According to Table 3 in the sinus/nasal cavity tumor location patient group, irradiation of the adapted plan started at a random week during the treatment course. The median replanning week for this group is week 4, with a range spanning from week 1 to week 7.

In the brain tumor location patient group, there were no patients who began the irradiation of the new plan beyond week 5. The median replanning week for this group is week 5, ranging from week 1 to week 5. Notably, 6 out of 11 brain tumor patients, i.e., over 50%, were replanned during week 5. The results indicate that the more critical period from the point of view of adaptive radiotherapy in CCB is the second half of the treatment cycle, regardless of the group of patients analyzed.

Table 4 and Table 5 present the results regarding the occurrence of improvements or deteriorations in dose distribution parameters. It is noteworthy that a significant improvement in dose distribution was observed in the PTV area, with enhancements seen in 80% or more of cases for each PTV parameter. The results are presented in two separate tables, distinguishing between patients with tumors in the sinus area and those with brain tumors. For the group with brain tumors, the dose assessment within the brain structure was omitted. This was due to the fact that the medical physicist did not include a brain structure in the excision of the PTV area during the planning process, meaning that any assessment would also have included the PTV area. Note that some of the OAR were not delineated during the plan preparation. For brain tumor irradiation, the spinal cord was not delineated in most cases (54.5%), resulting in lower quality statistical analysis. In the case of proton radiotherapy, we are dealing with a much lower level of scattered radiation. Irradiation is more conformal, and consequently, organs located several centimeters from the target area are practically unaffected (if they are not in the path of the proton beam). Consequently, the physician decides not to contour some of the structures.

Table A3 in Appendix A shows a catalog of OAR and PTV parameters assessed in the analysis for the entire group of patients (without separating sinus and brain tumors), along with the number of patients exhibiting improvement, worsening or no change, considering the uncertainty in parameter determination. An analysis of Table A3 shows a clear statistically significant improvement in the PTV area, for all parameters analyzed.

**Figure 3:**
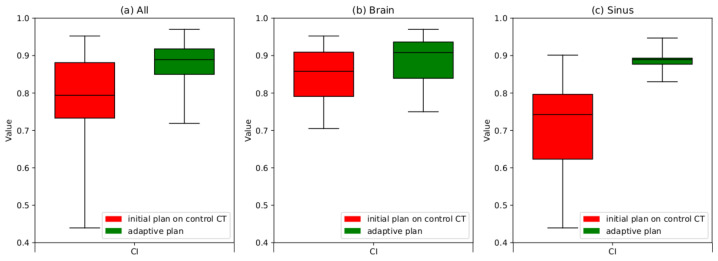
Boxplots illustrating the CI following the implementation of the adaptation procedure. The boxes extend from the Q1 to Q3 quartile values of the data, with a line at the median value. Red boxes signify the outcomes derived from recalculating the initial plan on control scans. Green boxes portray the outcomes achieved after adapting the plan to the current anatomical conditions of the patient (replan); (**a**) CI results for the entire patient group, (**b**) CI results for patients exclusively with brain tumors, (**c**) CI results for patients with tumors in the sinus and nasal cavities.

**Figure 4:**
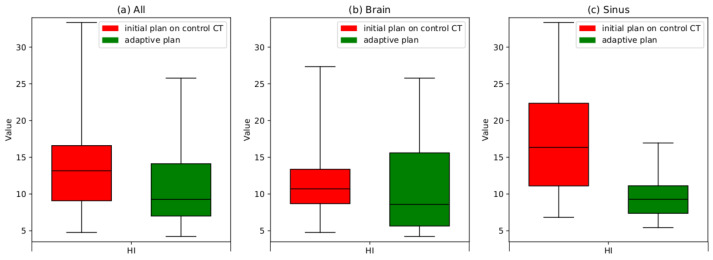
Boxplots illustrating the changes in HI following the implementation of the adaptation procedure. The boxes extend from the Q1 to Q3 quartile values of the data, with a line at the median value. Red boxes represent the outcomes obtained after recalculating the original plan on control scans. Green boxes represent the results obtained after adapting the plan to the patient’s current anatomical conditions (replan); (**a**) HI results for the entire patient group, (**b**) HI results for a group of patients with brain tumors only, (**c**) HI results for a group of patients with sinus and nasal cavities tumors.

**Figure 5:**
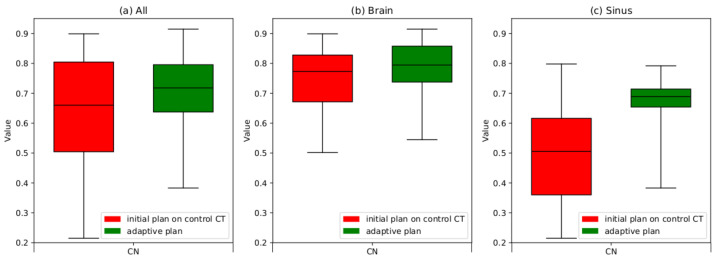
Boxplots illustrating the changes in the CN after applying the adaptation procedure was applied. The boxes extend from the Q1 to Q3 quartile values of the data, with a line at the median value. Red boxes represent the results obtained after recalculating the initial plan into control scans. Green boxes represent the results obtained after adapting the plan to the patient’s current anatomical conditions (replan); (**a**) the results of the CN for the whole group of patients, (**b**) the results of the CN for a group of patients with brain tumors only, (**c**) the results of the CN for a group of patients with sinus or nasal cavity tumors.

The results obtained for the CI, HI and CN indicate improvement resulting from the APT. A statistical hypothesis test for the parameters specified for the PTV demonstrates that the preparation of an adaptive plan enhances the dose distribution both in the treated area (PTV) with the statistical significance at α = 0.05. However, the statistical hypothesis test for the parameters specified for selected OARs leads to the conclusion that there is no statistically significant evidence, at α = 0.05. A qualitative evaluation of the enhancement in dose distributions within the PTV area is most conveniently observed through the block diagrams presented in Figure 3, Figure 4 and Figure 5.

Based on the results presented in the graphs in Figure 3, it is apparent that the adaptive procedure has played a pivotal role in elevating the quality of the treatment plan. The mean CI parameter exhibited an improvement of approximately 15% across the entire patient group post the adaptive procedure. Particularly noteworthy is the more substantial improvement observed in patients with tumors in the sinus and pharynx region, amounting to approximately 28%. In contrast, patients with brain tumors demonstrated an improvement of about 4%.

According to Figure 4, the average HI parameter for the entire patient group showed an improvement of approximately 22% following the adaptive procedure. Similarly, patients with tumors in the sinus and pharynx area demonstrated a more significant improvement, reaching about 35%. An improvement of approximately 10% was in a group of patients with brain tumors.

Based on Figure 5, the average value of the CN parameter for the entire examined patient group increased by approximately 24%. In the case of patients with tumors in the sinuses and nasal cavities, the improvement in the abovementioned average value reached 44%, while for patients with brain tumors, there was an improvement of about 5%. A significant reduction (>30%) in the standard deviation was clearly observed for the group of patients with tumors in the sinus and nasal area. This indicates that the quality of the dose distribution was similar for each of the analyzed cases.

**Figure 6:**
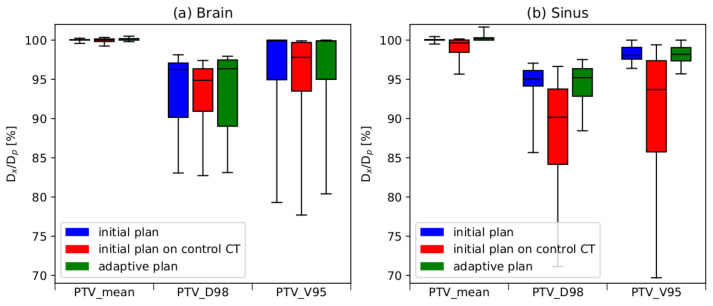
Boxplots illustrating the DVH parameters ($D_{mean}$, $D_{98\%}$, $V_{95\%}$) within the PTV for patients with tumors located in the brain (**a**) and patients with cancer within the sinus and nasal areas (**b**). The boxes extend from the Q1 to Q3 quartile values of the data, with a line at the median value.

Figure 6 shows the feature boxplots for various parameters within the PTV range, encompassing the mean dose, $D_{98\%}$, and $V_{95\%}$, specifically for the two distinct groups of patients.

The obtained results lead to the conclusion that in a significant majority of the analyzed cases, the adaptive procedure enables the restoration of dose parameters to levels closely resembling the nominal values for the PTV. As depicted in Figure 6, the recalculation of the original plan to the control CT leads to a deterioration in dose distribution in both analyzed patient groups. Significantly greater differences were noted in the group of patients with tumors located in the sinus and nasal cavities. Within this particular patient cohort, anatomical changes and variations in sinus filling had a more pronounced impact on the quality of the dose distribution.

## 4. Discussion

In this study, we evaluated the dosimetric impact of a clinically applied APT protocol on 21 H&N tumor patients, who represent 11.7% of the 116 patients treated with APT at the CCB proton therapy center between 2020 and mid-2024. Most patients with APT have tumors located in the H&N area (∼90%). APT led to improved target coverage and either similar or slightly reduced doses to OARs. When no plan adaptation was performed, target coverage fell below clinical goals, but this was restored after the application of APT. A single APT was, in most cases, sufficient to restore target coverage. Based on the available data, the second half of the treatment cycle is the more critical period for adaptive radiotherapy at CCB, regardless of the patient group analyzed.

The findings obtained in this research are in line with results from other institutions [15,16,17]. One of the strengths of this study is the number of patients that have undergone retrospective analysis, as in the majority of publications, the number of analyzed cases did not exceed 10 patients [16]. However, gathering a sufficient number of replanning cases for specific indications and treatment planning protocols—such as tumor size, location and number of fields—remains challenging. To confirm the findings presented here, we are actively extending the existing database, prospectively collecting data of patients that have undergone APT. Unifying the APT protocols and combining data from multiple institutions would further reinforce the evidence of APT. Moreover, this study indicates that the motivation for preparing an adaptive plan originated from both the disturbance in the dose distribution in the target and OARs [1,18]. Our data show that adapting the dose distribution in the target area typically improves dose distribution in the OARs region. This is contradictory to the majority of publications on APT, where the primary trigger for APT was the deterioration of the dose in the target volume only [15,17]. 

The great advantage of the presented analysis is that the presented data were used for APT at an operating proton therapy facility. Our findings show that the replanning procedure typically takes about five days. The current protocol involves multiple steps: medical decision-making, contour delineation, treatment plan reoptimization and dosimetric verification. While the protocol could be enhanced by incorporating AI to support medical decisions, automating contouring [19,20], replanning in the TPS [21] and using Monte Carlo-based dosimetric verification [22,23], it reflects legal requirements that do not differentiate between original treatment planning and treatment plan adaptation. Currently, the collaborative effort of the RAPTOR consortium is dedicated to advancing proton therapy by developing and implementing automative, online treatment plan adaptation strategies to improve precision and outcomes in cancer treatment. Online APT could offer substantial advantages. As indicated in [16], online daily APT allows for the quickest response to changes in patient anatomy. As it stands, APT at the CCB Krakow proton center demands significant manpower and technical resources, which must be weighed against the clinical benefits for the patient. Notably, even with offline APT, the protocol still leads to dosimetric improvements over no APT, validating the current clinical approach. 

One crucial aspect of APT is the frequency of control imaging. More frequent control imaging would help monitor daily anatomical changes and assess target dose deterioration, aiding in the optimal timing for offline APT. This approach could also determine if and when online APT is advantageous, considering the patient’s exposure to daily imaging doses. Significant anatomical changes with high variability are most likely in tumors located in perisinusoidal areas. In contrast, tumors in locations such as the spine, brain or sacrum may experience less dynamic changes due to the radiation therapy process itself. Therefore, a thorough analysis of the benefits and drawbacks of both online and offline APT, especially in relation to patient imaging dose, is essential. 

In summary, the shortcomings of this study, which are planned to be considered in the future, relate either to patient data collection and analysis or the clinical APT protocol that is currently applied in the CCB Krakow proton center. In terms of this study, increasing patient statistics would reduce the uncertainty of the results and could provide much more sound conclusions that may be the basis for correction of the existing APT protocol. As discussed above, increased patient statistics would enable the division of the patient cohort into smaller, more concise sub-groups, e.g., according to tumor size, location, tumor type, treatment dose and the number of irradiation fields, frequency of control imaging or patient age, etc. Addressing these shortcomings and expanding the patient dataset will be crucial for refining the APT protocol, leading to more precise and individualized treatment strategies in proton therapy.

## 5. Conclusions

We studied the treatment plan adaptation protocol applied clinically at the CCB Krakow proton therapy center for a cohort of 21 selected patients treated between 2020 and mid-2024. We evaluated dose distributions before and after plan adaptation using original and control CT scans. The examination of individual DVH parameters and the conducted statistical tests collectively affirm that the quality of the adapted plans has improved compared to the initial plans, especially regarding the coverage of the PTV area. While the reduction in the dose delivered to critical organs was a common objective of the adaptation procedure, the statistical tests indicate no significant differences in critical organ exposure between the initial and adapted plans. This study justifies the application of the offline APT in the CCB Krakow proton therapy center. 

## Figures and Tables

**Table 1:** The overall statistics of patients treated at CCB with proton beam scanning by year.

Year	Number of Patients with APT	Total Number of Patients Treated in CCB	Fraction of APT Patients
2020	14	133	10.5%
2021	8	171	4.7%
2022	20	177	11.3%
2023	38	312	12.2%
mid.2024	36	200	18.0%
**sum:**	**116**	**993**	**11.7%**

**Table 2:** Description of the parameters employed in the evaluation of the plans.

Parameter	Description	Evaluated for
$D_{\max}$, $D_{\mathrm{mean}}$ [Gy]	Maximal/mean dose within the organ structure	Brain, chiasm, optic nerves, brainstem, spinal cord, pituitary, cochleas and PTV
$D_{\mathrm{near}\_\max}$ [Gy]	Dose delivered to 0.1 ${\mathrm{cm}}^{3}$ of a structure volume	Brain, chiasm, optic nerves, brainstem, spinal cord, pituitary, cochleas
$V_{95\%}$ [%]	Volume of organ receiving at least 95% of prescribed dose	PTV
$D_{98\%}$ [Gy]	Dose received by 98% of the organ volume	PTV

**Table 3:** Statistics on the initiation of adaptive plan irradiation for sinus/nasal cavity and brain tumor patient groups in proton radiotherapy.

Tumor Location Group	No of Patients Started Irradiation of the Adapted Plan at:							Sum of Patients	Median
	Week 1	Week 2	Week 3	Week 4	Week 5	Week 6	Week 7		
**Sinus/nasal** ** cavity**	2	1	1	2	1	2	1	10	week 4
**Brain**	2	1	0	2	6	0	0	11	week 5

**Table 4:** A catalog of OARs and PTV parameters assessed in the analysis, along with the number of sinus/nasal area cancer patients exhibiting improvement, worsening or no change, considering the uncertainty of accuracy in parameter determination. The final column includes the outcomes from the nonparametric statistical hypothesis test.

OAR or PTV Parameter		No. of Patients			Wilcoxon Signed-Rank Test	
		Improving	Worsening	No Significant Change	*p*-Value	Test Result *
Brain	$D_{\max}$	7	3	0	0.064	*✗*
	$D_{\mathrm{near}\_\max}$	4	4	2	0.432	
Brainstem	$D_{\max}$	4	5	1	1.000	*✗*
	$D_{\mathrm{near}\_\max}$	5	3	2	1.000	
Chiasm	$D_{\max}$	4	4	2	0.846	*✗*
	$D_{\mathrm{near}\_\max}$	5	3	2	0.375	
Optic Nerve L	$D_{\max}$	6	3	0	0.084	*✗*
	$D_{\mathrm{near}\_\max}$	5	4	0	0.432	
Optic Nerve R	$D_{\max}$	6	3	0	1.000	*✗*
	$D_{\mathrm{near}\_\max}$	5	2	2	0.359	
Spinal Cord	$D_{\max}$	6	2	1	0.301	*✗*
	$D_{\mathrm{near}\_\max}$	4	4	1	0.820	
PTV	$D_{\mathrm{mean}}$	8	0	2	0.006	*✓*
	$D_{98\%}$	8	2	0	0.020	*✓*
	$V_{95\%}$	8	2	0	0.014	*✓*
	CI	8	2	0	0.027	*✓*
	CN	8	1	1	0.049	*✓*
	HI	9	1	0	0.004	*✓*

* ✓—statistically significant improvement, ✗—no statistical evidence of improvement.

**Table 5:** A catalog of OARs and PTV parameters assessed in the analysis, along with the number of brain area tumor patients exhibiting improvement, worsening or no change, considering the uncertainty of accuracy in parameter determination. The final column includes the outcomes from the nonparametric statistical hypothesis test.

OAR or PTV Parameter		No. of Patients			Wilcoxon Signed-Rank Test	
		Improving	Worsening	No Significant Change	*p*-Value	Test Result *
Brainstem	$D_{\max}$	5	1	5	0.054	*✗*
	$D_{\mathrm{near}\_\max}$	5	2	4	0.320	
Chiasm	$D_{\max}$	3	5	3	0.278	*✗*
	$D_{\mathrm{near}\_\max}$	5	3	3	0.508	
Optic Nerve L	$D_{\max}$	2	4	4	0.492	*✗*
	$D_{\mathrm{near}\_\max}$	3	2	5	0.767	
Optic Nerve R	$D_{\max}$	5	2	3	0.275	*✗*
	$D_{\mathrm{near}\_\max}$	3	4	3	0.846	
Pituitary	$D_{\max}$	5	4	0	0.496	*✗*
	$D_{\mathrm{near}\_\max}$	6	3	0	0.164	
Cochlea L	$D_{\max}$	4	3	2	0.250	*✗*
	$D_{\mathrm{near}\_\max}$	6	0	3	0.012	*✓*
Cochlea R	$D_{\max}$	6	3	0	0.301	*✗*
	$D_{\mathrm{near}\_\max}$	6	3	0	0.250	
PTV	$D_{\mathrm{mean}}$	7	1	3	0.365	*✗*
	$D_{98\%}$	9	2	0	0.275	*✗*
	$V_{95\%}$	9	1	1	0.019	*✓*
	CI	8	0	3	0.007	*✓*
	CN	7	1	3	0.017	*✓*
	HI	9	2	0	0.175	*✗*

* ✓—statistically significant improvement, ✗—no statistical evidence of improvement.

## Data Availability

The datasets generated and/or analyzed during the current study are accessible from the main author upon reasonable request, contingent on the consent of all co-authors, and given that there are no additional costs associated with data sharing.

## Abbreviations

The following abbreviations are used in this manuscript:

APT	adaptive proton therapy
CCB	Cyclotron Centre Bronowice
CT	computed tomography
CTV	clinical target volume
DVH	dose-volume histogram
H&N	head and neck
IMPT	intensity modulated proton therapy
OAR	organ at risk
PBS	pencil beam scanning
PTV	planning target volume
RBE	relative biological effectiveness
RTT	radiation therapist

## Appendix A

Table A3 shows a catalog of OAR and PTV parameters assessed in the analysis for the entire group of patients (without separating sinus and brain tumors), along with the number of patients exhibiting improvement, worsening, or no change, considering the uncertainty in parameter determination. The final column presents the results of the nonparametric statistical hypothesis test.

Table A1 and Table A2 listing patients chosen for the study on proton therapy adaptation of perisinusoidal and brain areas at the Cyclotron Centre Bronowice in Krakow divided into two groups, along with tumour locations and treatment plan details.

**Table A1:** List of patients with tumors located in the sinus/nasal area selected for the study, including treatment plan specifications. In the column listing the number of fractions completed for the initial plan, information was added in parentheses indicating the week of treatment when the adaptive plan was introduced. Radiation therapy is performed daily, 5 days a week.

Patient No.	Age	Tumour Location	Days after CT Reskan to Adapted Plan	Original PTV [cm^3^]	Adapted PTV [cm^3^]	No. of Fields	Fraction Dose [GyRBE]	No of Fractions with Original Plan/All Delivered Fractions	Replan Motivation	Radiotherapy Combined with:
1	38	Sinus cavity	4	31.66	31.29	3	1.8	19/30 (week 4)	tumor regression	recurrence
2	76	Nasal cavity	7	211.11	211.55	6	2	24/35 (week 5)	dose increase in OARs	immunotherapy
3	41	Nasal cavity	3	60.48	60.11	6	2	16/33 (week 4)	dose increase in OARs	surgery
4	35	H&N Nasal cavity +lymph nodes	6	274.95	235.35	4	2	26/35 (week 6)	tumor regression	chemotherapy
5	43	Sinus cavity	the same CT	305.45	370.88	6	2	14/35 (week 3)	tumor progression	chemotherapy
6	69	H&N Sinus cavity	7	850.81	923.88	6	2	8/30 (week 2)	tumor progression	chemotherapy
7	59	Sinus cavity	5	165.84	165.35	6	1.8	31/37 (week 7)	dose decrease in PTV	chemotherapy
8	70	H&N Sinus cavity +lymph nodes	4	107.66	107.93	6	2	27/35 (week 6)	disturbance of dose distribution in PTV nad OARs	chemotherapy
9	57	H&N. Sinus cavity	the same CT	453.15	453.15	4/5 *	2	2/27 (week 1)	dhange of the dose constrains for OAR	surgery and chemotherapy
10	45	H&N Nasal cavity +lymph nodes	the same CT	197.32	197.32	6	2	2/35 (week 1)	dhange of the dose constrains for OAR	chemotherapy

* 4 fields in initial plan, 5 fields in APT.

**Table A2:** List of patients with tumors located in the brain selected for the study, including treatment plan specifications. In the column listing the number of fractions completed for the initial plan, information was added in parentheses indicating the week of treatment when the adaptive plan was introduced. Radiation therapy is performed daily, 5 days a week.

Patient No.	Age	Tumour Location	Days after CT Reskan to Adapted Plan	Original PTV [cm^3^]	Adapted PTV [cm^3^]	No. of Fields	Fraction Dose [GyRBE]	No of Fractions with Original Plan/All Delivered Fractions	Replan Motivation	Radiotherapy Combined with:
1	37	Brain	the same CT	148.46	148.46	4	1.8	2/30 (week 1)	Dose increase in OARs	surgery
2	21	Brain	6	588.55	589.21	6	1.8	2/33 (week 1)	weight gaining —disturbance of dose distribution in OARs	surgery
3	60	Brain	6	352.13	350.17	3	1.8	24/30 (week 5)	tumor regression	surgery
4	38	Brain	1	152.24	150.77	2	1.8	22/30 (week 5)	tumor regression	surgery
5	30	Brain	the same CT	663.92	663.92	3	1.8	6/30 (week 2)	dhange of the dose constrains for OAR	surgery
6	48	Brain	14	232.24	231.20	3	1.8	22/30 (week 5)	disturbance of dose distribution in OARs	surgery
7	44	Brain	6	365.21	363.81	2	1.8	23/33 (week 5)	increased Dmax to Brainstem	-
8	71	Brain	2	132.45	131.77	3	1.8	22/30 (week 5)	disturbance of dose distribution in CTV	-
9	40	Brain	7	818.47	834.13	4	1.8	19/33 (week 4)	tumor progression	surgery
10	34	Brain	6	539.8	537.31	3	1.8	22/30 (week 5)	increased Dmax to Brainstem	surgery
11	19	Brain	3	673.84	671.51	3	1.8	22/30 (week 4)	increased Dmax to Brainstem and Chiasm	surgery

**Table A3:** List of OAR and PTV parameters evaluated simultaneously for both groups of patients.

OAR or PTV Parameter		No. of Patients			Wilcoxon Signed-Rank Test	
		Improving	Worsening	No Significant Change	*p*-Value	Test Result *
Brainstem	$D_{\max}$	9	6	6	0.179	*✗*
	$D_{\mathrm{near}\_\max}$	10	5	6	0.038	
Chiasm	$D_{\max}$	7	9	5	0.355	*✗*
	$D_{\mathrm{near}\_\max}$	10	6	5	0.313	
Optic Nerve L	$D_{\max}$	8	7	4	0.277	*✗*
	$D_{\mathrm{near}\_\max}$	8	6	5	0.421	
Optic Nerve R	$D_{\max}$	11	5	3	0.395	*✗*
	$D_{\mathrm{near}\_\max}$	8	6	5	0.595	
PTV	$D_{\mathrm{mean}}$	15	1	5	0.003	*✓*
	$D_{98\%}$	17	4	0	0.007	*✓*
	$V_{95\%}$	17	3	1	0.001	*✓*
	CI	16	2	3	0.001	*✓*
	CN	15	2	4	0.002	*✓*
	HI	18	3	0	0.001	*✓*

* ✓—statistically significant improvement, ✗—no statistical evidence of improvement.
